# The Effect of Environmental Enrichment on Laboratory Rare Minnows (*Gobiocypris rarus*): Growth, Physiology, and Behavior

**DOI:** 10.3390/ani12040514

**Published:** 2022-02-18

**Authors:** Chunsen Xu, Miaomiao Hou, Liangxia Su, Ning Qiu, Fandong Yu, Xinhua Zou, Chunling Wang, Jianwei Wang, Yongfeng He

**Affiliations:** 1 Institute of Hydrobiology, Chinese Academy of Sciences, Wuhan 430072, China; 18227588944@163.com (C.X.); houmiao006@163.com (M.H.); suliangxia027@126.com (L.S.); qiuning181@163.com (N.Q.); yufd666@163.com (F.Y.); 18370085282@163.com (X.Z.); chwang@ihb.ac.cn (C.W.); 2 University of Chinese Academy of Sciences, Beijing 100049, China; 3 National Aquatic Biological Resource Center, Institute of Hydrobiology, Chinese Academy of Sciences, Wuhan 430070, China

**Keywords:** rare minnow, environmental enrichment, growth, physiology, anxiety-like behavior, welfare

## Abstract

**Simple Summary:**

Environmental enrichment is an important part of animal welfare. In this study, rare minnow in different rearing conditions underwent comprehensive evaluation regarding growth, anxiety-like behavior, and physiology parameters. Results showed that there were no differences in SGR, anxiety-like behavior, DA, DOPAC, and 5-HIAA levels between control and enriched groups. However, the enriched group had higher cortisol and 5-HT levels. Therefore, researchers should focus on the effect of environmental enrichment regarding the welfare of rare minnow and how it effects the validity of data from laboratory studies.

**Abstract:**

Environmental enrichment is a method to increase environmental heterogeneity, which may reduce stress and improve animal welfare. Previous studies have shown that environmental enrichment can increase the growth rate, decrease aggressive and anxiety-like behaviors, improve learning ability and agility, and reduce cortisol levels in animals. These effects usually differ between species. Unfortunately, habitat enrichment on laboratory fish is poorly studied and seldom adopted in care guidance. Rare minnows (*Gobiocypris rarus*) have been cultured as a native laboratory fish in China in barren banks without environmental enrichment since 1990; they have been widely used in studies on ecotoxicology, environmental science, and other topics. The purpose of this study was to investigate the effect of environment enrichment on the growth, physiological status, and anxiety-like behavior of laboratory rare minnows. We observed and analyzed SGR, cortisol levels, DA, DOPAC, 5-HT and 5-HIAA, and anxiety-like behavior indexes after one month of treatment in barren (control) and enrichment tanks. We found that there were no significant differences in SGR, anxiety-like behavior, DA, DOPAC, or 5-HIAA levels between the two treatments. However, higher cortisol and 5-HT levels were observed in the enrichment tanks. This study suggests that rare minnows might be influenced by their living environment, and future related studies should consider their environmental enrichment.

## 1. Introduction

The physical and mental status of animals is often affected by the environment. In fact, a lack of shelter in artificial environments may elicit a variety of stress responses in fish, including physiological, psychological, and behavioral effects [1]. In addition, environment homogeneity may decrease fishes’ learning opportunities and learning ability [1]. Enrichment is a method to increase environmental heterogeneity and provide shelter by introducing plants, cobbles, and other physical structures into the rearing environment [1,2].

As a result of simulating the natural habitat, enrichment usually has a positive impact on fish because it provides the fish diverse physical or hydraulic environments and opportunities to choose its habitat. Many studies have shown that environmental enrichment can enhance growth rate [3,4,5,6,7], decrease aggressive and anxiety-like behaviors [8,9,10], enhance learning ability [11,12,13] and agility [14], and reduce cortisol (an indicator of stress) levels [8]. However, some studies have identified cases in which enrichment elicited negative or neutral effects [15,16,17,18,19,20,21]. The differences in these results are not only related to differences in species or life history stages, but also with the type and level of enrichment [2,22].

Owing to their small size, high fecundity, short generation, rapid sexual maturity, and simple facility, laboratory fish are widely used in the life sciences [23]. The welfare of laboratory fish is becoming a new and urgent topic and, as it is an important part of animal welfare, environmental enrichment of laboratory fish should be given more attention. Unfortunately, environmental enrichment is less adopted in care guidance instructions for laboratory fish [21]. For instance, studies have shown that environmental enrichment has certain benefits, such as reducing zebrafish anxiety-like behavior and increasing proliferating cell nuclear antigen positive nuclei and spatial learning ability [10,15,24], but most laboratories still use barren tanks to raise zebrafish. Enriching laboratory fish not only improves the fish’s welfare [22] but may also impact the validity of the data collected [25,26] and feeding management [27].

The rare minnow (*Gobiocypris rarus*) is a small cyprinid fish that is widely used as a native laboratory fish for chemical testing and research on disease, toxicology, behavior, and genetics [28,29,30,31,32,33,34]. For its use as a laboratory animal, serial standard drafts have been established on topics including controls on pathogens, heredity, environment, and nutrition. However, environmental enrichment is not considered essential for animal welfare, although previous study showed rare minnow had a significant preference for an enriched environment [35]. The aim of this study was to investigate the effect of environmental enrichment on the growth, anxiety-like behavior, and physiological status of laboratory rare minnows.

## 2. Material and Methods

### 2.1. Fish Culturing and Environmental Enrichment

**Figure 1:**
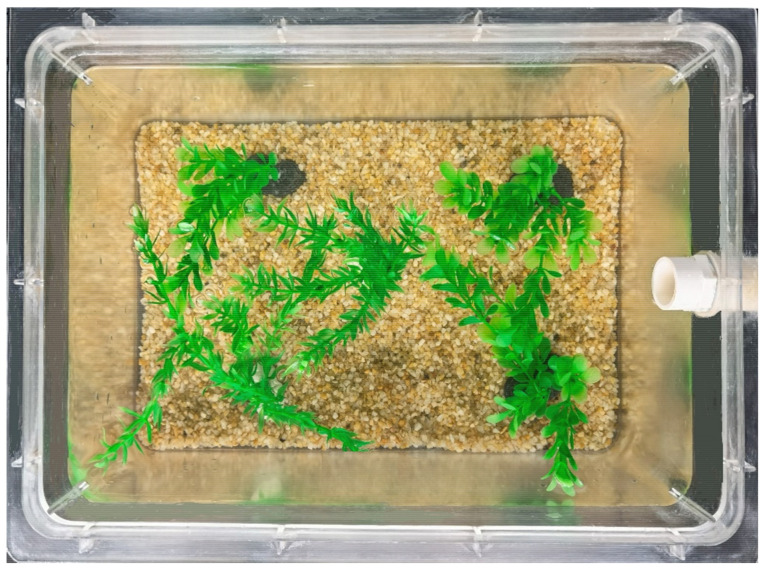
Photographs of the enrichment group.

Rare minnows (*Gobiocypris rarus*) were provided by the National Aquatic Biological Resource Center (NABRC). The whole experiment was done in NABRC’s laboratory. In order to ensure animal welfare and meet the sampling requirements, 72 adult fish aged 6 months with an average total length of 33.5 ± 2.44 mm and body weight of 0.39 ± 0.05 g were randomly, equally placed into six plastic tanks (length: 40.0 cm, width: 25.0 cm, height: 20.0 cm), exhibiting 12 rare minnows per tank. All individuals were in the same recirculating aquatic housing system equipped with multistage filtration including activated filter stone, filter sponge, and UV sterilization. The tanks were arranged into two treatments: environmental enrichment and control. In the three enriched replicate tanks, the whole bottom was covered with faint yellow gravel, ~1 cm deep and ~0.4 cm in diameter, and plastic plants (three artificial plants that mimic *Potamogeton perfuliatus*, signal leaf length: 2.2 cm, plant length: 16 cm, color: green). Two artificial plants mimicking *Hydrilla verticillata* (signal leaf length: 1.5 cm, plant length: 16 cm, color: green) were added to occupy approximately 40% of the surface area (Figure 1). The other three barren replicate tanks without any enrichment were set as the control group. Test fish were reared in the tanks for one month until they were sampled or used for the behavior experiments.

During the rearing period, we maintained the water depth at 16 cm and the water flow rate at 700 mL/min. The light/dark cycle was 12:12 h. Water temperature was maintained at 25.6 ± 0.2 °C. The pH was 7.49–7.80. Fish were fed commercial dry pellets (crude protein ≥ 35%, crude fat ≥ 3.0%, crude fiber ≤ 8.0%, crude ash ≤ 15%, moisture ≤ 10%, calcium ≥ 12%, phosphorus ≥ 0.6%, lysine ≥ 1.5%) twice daily at 10:00 a.m. and 4:00 p.m. No injuries or deaths were found during the experiment, and the remaining individuals were transferred back to the NABRC after the experiment ended.

### 2.2. Sampling and Measuring of Physiological Parameters

At the end of the month, four fish from the same tanks (three replicates enriched and control tanks) were euthanized in ice water [36]. Immediately after the total body length and body weight were measured, each fish was dissected on ice. The body weight and body length of the remaining fish were measured after the anxiety-like behavior test. All the brain tissue was used to determine the levels of neurotransmitters, including Dopamine (DA), Serotonin (5-HT), 3,4-dihydroxyphenylacetic acid (DOPAC), 5-hydroxyindoleacetic acid (5-HIAA), and brain protein. The rest of the body was used to determine the cortisol level. All procedures and the experimental protocols were approved by the Institutional Animal Care and Use Committee of the Institute of Hydrobiology, Chinese Academic of Sciences (IHB/LL/2020025).

Four fish brains from the same tank were mixed into one sample and two bodies from the same tank were mixed into one sample. Samples of brain or body were homogenized in cold PBS (9× weight, pH 7.4) and centrifuged at 3000 rpm for 20 min in a refrigerated centrifuge (4 °C). The supernatants were collected and used for analysis. The cortisol, DA, DOPAC, 5-HT, and 5-HIAA levels were measured using a fish-specific commercial ELISA Assay Kit (Jiangsu Meimian Industrial Co., Ltd., Yancheng, China) according to the manufacturer’s guidelines. Brain protein was measured using a commercial BCA Assay Kit (Jiangsu Meimian Industrial Co., Ltd., Yancheng, China) according to the manufacturer’s guidelines.

### 2.3. Anxiety-like Behavioral Studies

#### 2.3.1. Light-Dark Test

**Figure 2:**
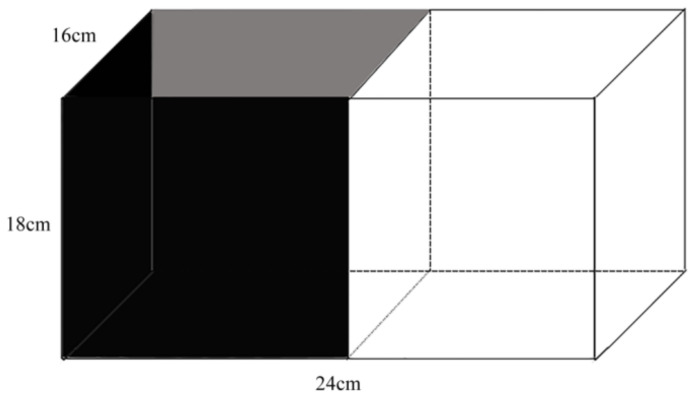
Diagram of the light–dark test tank (24 cm × 16 cm × 18 cm high, water level is 10 cm).

The light-dark test was used for the anxiety-like behavior test in fish [10,37]. The light-dark experiment was designed based on a previous study [10], based on scototaxis. Rare minnows prefer a dark environment and are suitable for this light-dark test. The reduction in the number of entries to the light part and the time spent in the light part were considered indicators of anxiety-like behavior [37]. A rectangular glass tank was divided into two equal size parts, which were affixed with a white or black sticker separately (Figure 2). The 12 enriched and 12 control fish (four from each replicate enriched and control tank) were randomly selected and observed one by one, with one enriched and one control fish observed simultaneously. Each fish was only observed once to prevent any impacts from multiple measurements. Test fish were gently put into the dark part of the light-dark tank. The fish were allowed to adapt to the tank for 2 min, then data were collected with a camera (TTQ, China, Resolution: 1920 × 1080) for 8 min. The number of entries to the light part and total time spent in the light part were counted manually by playback video. In order to minimize human interference, we did not set up any partitions during the adaptation period. Fresh system water was added after each trial.

#### 2.3.2. Novel Tank Test

**Figure 3:**
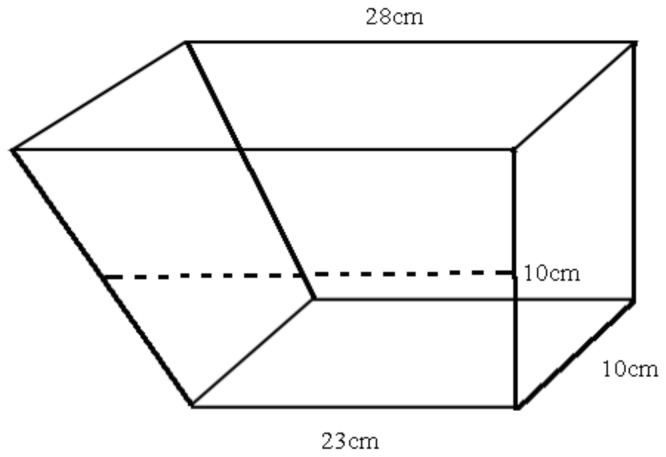
Front view of the novel tank test. Water level is 10 cm.

The novel tank test was used for anxiety-like behavior test in fish, and mainly utilized their natural instinct to dive to the bottom of the tank and explore its environment [38,39]. The apparatus consisted of a trapezoidal glass tank (Figure 3). The tank was divided into two parts of the same height: a bottom layer and an upper layer. Except for the front camera (TTQ, China, Resolution:1920 × 1080), all parts of the test tank were covered with white frosted stickers to prevent any external interference. All behavior parameters were counted manually by playback video.

The 12 enriched and 12 control naïve fish (four from each replicate enriched and control tanks) were randomly selected, and one fish was gently put into the tank for each test at the same time. The fish were allowed to adapt to the tank for 2 min, then data were collected with a camera for 8 min. Each fish was only observed once to prevent the impact of multiple measurements. Different fish were used for the different behavioral tests. The tank was filled with system water, which was replaced at the end of each trial. The behavior parameters included the number of entries to the upper layer and total time spent in the upper layer. The reduction in the number of entries to the upper layer and the time spent in the upper layer are considered indicators of anxiety [39].

### 2.4. Data Analysis

The specific growth rate (SGR) was used to evaluate growth. SGR% = 100 × (ln (BW_f_) − ln (BW_i_))/T, where BW_i_ represents initial body weight in mg, BW_f_ represents final body weight in mg; T(d) represents experimental period in days. DA, DOPAC, 5-HT, and 5-HIAA levels were normalized to total brain protein weight (stated as ng/g of brain protein) and cortisol levels were normalized to body weight (stated as ng/g body weight).

The Shapiro–Wilk Test was used to determine whether the data obey normal distribution. If the data met the assumption of normal distribution, then an independent samples *t* test was used to analyze the differences between the two groups. If the data did not meet the assumption of normal distribution, then a Mann-Whitney U test was used to analyze the differentiation between the two groups. There were no data excluded. All statistical analyses were performed using SPSS 25.0. Significance level was set at *p* < 0.05, and extremely significant level set at *p* < 0.001.

## 3. Results

### 3.1. Growth

**Figure 4:**
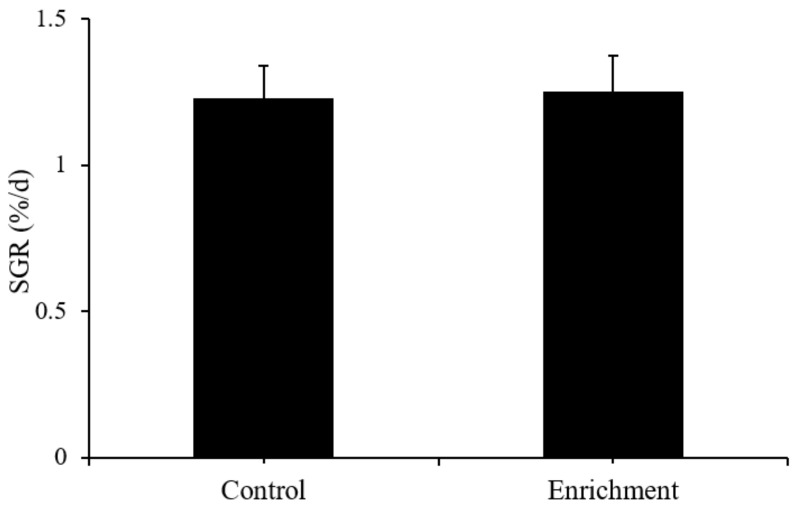
Special growth rate of body weight (SGR, mean ± S.D.) in different treatments (*n* = 3).

After one month of treatment in different environments, there was no significant difference between the two treatments (t = −0.1, *p* = 0.924) (Figure 4 and Table 1).

### 3.2. Physiological Parameters

**Figure 5:**
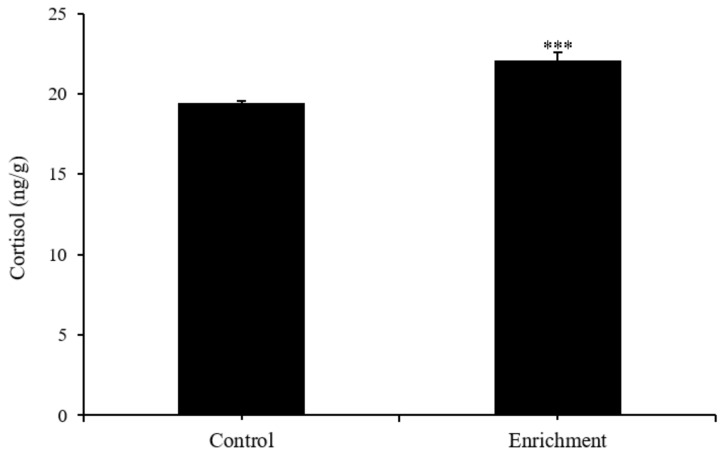
Cortisol level (mean ± S.D.) of the different environmental treatments (*n* = 3, three pooled samples of two fish each from each treatment). “***” represents extremely significant difference (*p* < 0.001).

**Figure 6:**
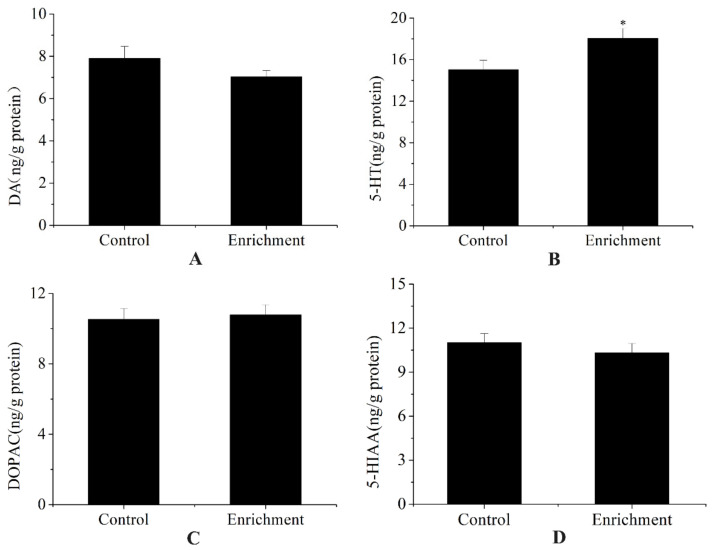
DA, DOPAC, 5-HT, and 5-HIAA levels (mean ± S.D.) of the different environmental treatments (*n* = 3, three pooled samples of four fish each from each treatment). (**A**) DA, (**B**) 5-HT, (**C**) DOPAC, and (**D**) 5-HIAA concentrations of the two groups. “*” represents a significant difference (*p* < 0.05).

Fish in the enriched treatment had significantly higher cortisol levels than fish in the control treatment (t = −12.93; *p* < 0.001) (Figure 5). There were no significant differences in DA (t = 1.923, *p* = 0.127), DOPAC (t = −0.432, *p* = 0.694), or 5-HIAA (t = 1.1, *p* = 0.335) levels between the two treatments, except that fish in the enriched treatment had higher 5-HT levels (t = −3.22; *p* = 0.032) (Figure 6 and Table 1). 

### 3.3. Anxiety-like Behavior

**Figure 7:**
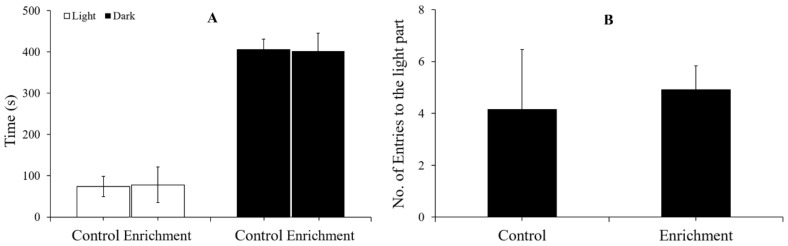
Time distribution and number of entries to the light part (mean ± S.E.) in the light-dark test (*n* = 12). (**A**) Time spent in the light and dark parts. (**B**) Numbers of entries to the light part.

No significant differences were found between the two treatments in terms of the time distribution between the light and dark areas (Mann–Whitney U test, U = 67, N = 12, *p* = 0.799) or number of entries to the light areas. (Mann–Whitney U test, U = 68.5, N = 12, *p* = 0.843) (Figure 7 and Table 2).

## 4. Discussion

**Figure 8:**
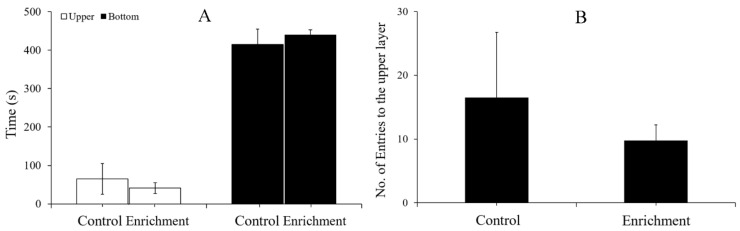
Time distribution and number of entries to the upper layer (mean ± S.E.) in the novel tank test (*n* = 12). (**A**) Time spent in the upper and bottom layer. (**B**) Numbers of entries to the upper layer.

In the novel tank test, there were no significant differences between the two treatments in the time distribution in the upper and lower layers of the tank (Mann–Whitney U test, U = 90, N = 12, *p* = 0.932) or the number of entries to the upper layer. (Mann–Whitney U test, U = 72, N = 12, *p* = 1) (Figure 8 and Table 2).

### 4.1. Effect of Enrichment on Growth

Growth is one of the most important physiological processes in the life history of fish. Any factor that affects animal feeding, digestion, activity, metabolism, and other physiological processes may affect animal growth. Growth is a simple index that reflects the comprehensive performance of many eco-physiological processes. When considering animal welfare, growth and survival are basic “natural demands”. If an animal’s growth is significantly affected by adverse conditions, then its welfare could deteriorate significantly [40]. Previous studies have shown that the factors affecting the growth of rare minnows include nutrients [41,42], water temperature [43] and other physical and chemical characteristics of their water environment, feeding, and other culturing strategies [43,44,45]. Therefore, these impact factors are restricted or controlled in the standard drafts of rare minnows to ensure their basic welfare. Although some studies revealed that environmental enrichment may help fish grow [3,4,5,6,7], our results showed no significant difference in SGR between the environmental enrichment and control fish, indicating that the barren tank (control) did not result in significant welfare deficiency. A small fish, the rare minnow seems to be well adapted to aquarium breeding. Rare minnow growth was not affected by complex environments; this may be one of the reasons why the species is used so widely as a laboratory animal.

### 4.2. Effect of Enrichment on Physiological Parameters

Poor conditions are often accompanied by stress, so stress level is a common metric for judging animal welfare. Stress, which usually refers to a change in one’s condition in response to a changing environment, is a physiological process of adapting to and maintaining homeostasis in the internal environment [46]. In the face of long-term stress, fish feeding, growth, immunity, and other factors suffer, and thus so does the fish’s welfare [40]. When fish encounter stress, their hypothalamic-pituitary-renal tissue (HPI) axis and neurotransmitter activity are generally considered to be the primary neuroendocrine regulators, and subsequent secondary reactions affect immune function, enzyme activity, blood parameters, etc., and finally lead to changes in behavior, reproductive ability, growth, and survival throughout the organism [47,48,49,50]. Brain serotonergic system activity, dopaminergic system activity, and cortisol levels are considered to be accurate and commonly used indicators of stress and welfare in fish [22,51,52,53,54]. Previous studies have shown that environmental enrichment may increase, decrease, or have no effect on fish cortisol levels [15,22,54,55,56,57]. In the present study, cortisol level in the environmental enrichment treatment did not decrease significantly. On the contrary, it was 22.07 (ng/g), 13% higher than in the control. This result is somewhat unexpected. It may be that the disturbances that resulted from removing artificial aquatic plants from the aquarium before euthanasia resulted in the higher cortisol level in the enrichment fish. On the other hand, hormonal response to stressors in general and chronic stressors in particular can be highly context dependent, and there is currently a lack of consensus as to the direction and magnitude of this response [58]. The reasons for the higher cortisol level in the enriched group need further investigation.

The dopaminergic system is usually correlated with reward and motor functions [59,60,61,62]. In the present study, there were no significant differences in DA and DOPAC levels between the control and environmental enrichment, indicating that environmental enrichment has no effect on the above-mentioned functions of rare minnows. This result is similar to that of the black rockfish (*Sebastes schlegelii*) [22].

The serotonergic system is usually involved in the regulation of various physiological functions in animals—such as aggressive behavior, anxiety, and depression—and plays especially important roles in emotion regulation [63,64,65,66]. For instance, rodent studies have demonstrated that serotonin depletion results in increased aggression, and serotonin augmentation results in decreased aggression [67]. Environmental enrichment rescued the depressive phenotype of female HD mice, corresponding with increased gene expression of specific 5-HT receptors in the hippocampus and cortex [65]. In the present study, significantly higher 5-HT levels were found in the environmental enrichment group, indicating that the enriched environment benefits the rare minnow.

### 4.3. Effect of Enrichment on Anxiety-like Behavior

When fish face environmental stress, their intuitive response is reflected in behavior. If the fish are unable to escape the threat, then their behavior may change. Enriched environments create a complex habitat that allows animals to choose suitable hiding places, thus protecting them from fear and stress. Studies have shown that environmental enrichment can reduce anxiety-like behaviors and improve wellbeing [10,68,69]. However, in the present study, there was no significant difference in the time distribution or the numbers of entries between the two treatments in either the light-dark or novel tank tests. In fact, rare minnows, which live in small water bodies gregariously in the wild, are easily domesticated for aquariums. Rare minnows reared in barren tanks rarely display panic behavior. For instance, when breeders step close to the aquarium, fish tend to approach—a sign that they are waiting to be fed—instead of hiding or escaping. Therefore, environmental enrichment might have a limited effect on anxiety-like behavior.

## 5. Conclusions

The present study showed that environmental enrichment has no significant effect on the growth, anxiety-like behavior, or dopamine level of rare minnow, but it does significantly affect 5-HT level and cortisol levels. In the present study, we used stone bedding and aquatic plants to simulate a natural environment. The bottom of the aquarium box was fully covered with stone bedding, and the coverage of aquatic plants reached 40%; these conditions were too inconvenient to use in daily cultures. In this study, whether the environment is enriched or not probably had a limited effect on fish growth and anxiety-like behaviors. However, higher 5-HT level might be benefiting the rare minnow. Therefore, the barren tank may not meet some of the welfare demands of the laboratory rare minnow. It is important to note that the type and level of environmental enrichment are diverse, and there might be different results. More research is needed to meet the welfare demands of the laboratory rare minnow; at the same time, it is also convenient for daily management.

## Figures and Tables

**Table 1:** Growth and physiology parameters of different rearing conditions.

	SGR (%/d)	Cortisol (ng/g)	DA (ng/g Protein)	DOPAC (ng/g Protein)	5-HT (ng/g Protein)	5-HIAA (ng/g Protein)
Control	1.13 ± 0.11	19.46 ± 0.11	7.9 ± 0.57	10.53 ± 0.62	15.03 ± 0.93	11.01 ± 0.62
Enrichment	1.25 ± 0.12	22.07 ± 0.5	7.03 ± 0.3	10.78 ± 0.58	18.06 ± 0.96	10.32 ± 0.64

**Table 2:** Anxiety-like parameters of different rearing condition.

	Light-Dark Test			Novel Tank Test		
	Light Time (s)	Dark Time (s)	No. of Entries	Upper Time (s)	Bottom Time (s)	No. of Entries
Control	74.17 ± 24.82	405.83 ± 24.82	4.17 ± 2.29	65.17 ± 40.43	414.83 ± 40.43	16.5 ± 10.23
Enrichment	77.92 ± 43.21	402.08 ± 43.21	4.92 ± 0.92	41.08 ± 14.41	438.92 ± 14.41	9.8 ± 2.45

## Data Availability

Data and associated calculation tools are available from the corresponding author upon reasonable requestion (wangjw@ihb.ac.cn).

## References

[1] Näslund J., Johnsson J.I. (2016). Environmental enrichment for fish in captive environments: Effects of physical structures and substrates. Fish Fish..

[2] Jones N., Webster M., Salvanes A. (2021). Physical enrichment research for captive fish: Time to focus on the DETAILS. J. Fish Biol..

[3] Kientz J.L., Barnes M.E. (2016). Structural complexity improves the rearing performance of rainbow trout in circular tanks. N. Am. J. Aquacult..

[4] Kientz J.L., Crank K.M., Barnes M.E. (2018). Enrichment of circular tanks with vertically suspended strings of colored balls improves Rainbow Trout rearing performance. N. Am. J. Aquacult..

[5] Crank K.M., Kientz J.L., Barnes M.E. (2019). An evaluation of vertically suspended environmental enrichment structures during Rainbow Trout rearing. N. Am. J. Aquacult..

[6] White S.C., Krebs E., Huysman N., Voorhees J.M., Barnes M.E. (2019). Use of suspended plastic conduit arrays during Brown Trout and Rainbow Trout rearing in circular tanks. N. Am. J. Aquacult..

[7] Voorhees J.M., Huysman N., Krebs E., Barnes M.E. (2020). Influence of water velocity and vertically-suspended structures on rainbow trout rearing performance. Open J. Anim. Sci..

[8] Zhang Z.H., Xu X.W., Wang Y.H., Zhang X.M. (2020). Effects of environmental enrichment on growth performance, aggressive behavior and stress-induced changes in cortisol release and neurogenesis of black rockfish Sebastes schlegelii. Aquaculture.

[9] Batzina A., Karakatsouli N. (2012). The presence of substrate as a means of environmental enrichment in intensively reared gilthead seabream Sparus aurata: Growth and behavioral effects. Aquaculture.

[10] Maximino C., de Brito T.M., Dias C.A.G.D., Gouveia A., Morato S. (2010). Scototaxis as anxiety-like behavior in fish. Nat. Protoc..

[11] Salvanes A.G.V., Moberg O., Ebbesson L.O.E., Nilsen T.O., Jensen K.H., Braithwaite V.A. (2013). Environmental enrichment promotes neural plasticity and cognitive ability in fish. Proc. R. Soc. B.

[12] Roy T., Bhat A. (2016). Learning and memory in juvenile zebrafish: What makes the difference—Population or rearing environment?. Ethology.

[13] Strand D.A., Utne-Palm A.C., Jakobsen P.J., Braithwaite V.A., Jensen K.H., Salvanes A.G.V. (2010). Enrichment promotes learning in fish. Mar. Ecol. Prog. Ser..

[14] Bergendahl I.A., Miller S., Depasquale C., Giralico L., Braithwaite V.A. (2017). Becoming a better swimmer: Structural complexity enhances agility in a captive-reared fish. J. Fish Biol..

[15] von Krogh K., Sorensen C., Nilsson G.E., Overli O. (2010). Forebrain cell proliferation, behavior, and physiology of zebrafish, Danio rerio, kept in enriched or barren environments. Physiol. Behav..

[16] White S.C., Barnes M.E., Krebs E., Huysman N., Voorhees J. (2018). Addition of vertical enrichment structures does not improve growth of three salmonid species during hatchery rearing. J. Mar. Biol. Aquacult..

[17] Brydges N.M., Braithwaite V.A. (2009). Does environmental enrichment affect the behavior of fish commonly used in laboratory work?. Appl. Anim. Behav. Sci..

[18] Näslund J., Rosengren M., Johnsson J.I. (2019). Fish density, but not environmental enrichment, affects the size of cerebellum in the brain of juvenile hatchery-reared Atlantic salmon. Environ. Biol. Fishes.

[19] Toli E.A., Noreikiene K., DeFaveri J., Merila J. (2017). Environmental enrichment, sexual dimorphism, and brain size in sticklebacks. Ecol. Evol..

[20] Woodward M.A., Winder L.A., Watt P.J. (2019). Enrichment increases aggression in zebrafish. Fishes.

[21] Wilkes L., Owen S.F., Readman G.D., Sloman K.A., Wilson R.W. (2012). Does structural enrichment for toxicology studies improve zebrafish welfare?. Appl. Anim. Behav. Sci..

[22] Zhang Z.H., Bai Q.Q., Xu X.W., Guo H.Y., Zhang X.M. (2020). Effects of environmental enrichment on the welfare of juvenile black rockfish Sebastes schlegelii: Growth, behavior and physiology. Aquaculture.

[23] Lawrence C. (2007). The husbandry of zebrafish (Danio rerio): A review. Aquaculture.

[24] Spence R., Magurran A.E., Smith C. (2011). Spatial cognition in zebrafish: The role of strain and rearing environment. Anim. Cogn..

[25] Williams T., Readman G., Owen S.F. (2009). Key issues concerning environmental enrichment for laboratory-held fish species. Lab. Anim..

[26] Killen S.S., Marras S., Metcalfe N.B., McKenzie D.J., Domenici P. (2013). Environmental stressors alter relationships between physiology and behaviour. Trends Ecol. Evol..

[27] Pounder K.C., Mitchell J.L., Thomson J.S., Pottinger T.G., Buckley J., Sneddon L.U. (2016). Does environmental enrichment promote recovery from stress in rainbow trout?. Appl. Anim. Behav. Sci..

[28] Su L.X., Xu C.S., Cai L., Qiu N., Hou M.M., Wang J.W. (2020). Susceptibility and immune responses after challenge with Flavobacterium columnare and Pseudomonas fluorescens in conventional and specific pathogen-free rare minnow (*Gobiocypris rarus*). Fish Shellfish Immunol..

[29] Wang J.J., Li P., Zhang Y.G., Peng Z.G. (2011). The complete mitochondrial genome of Chinese rare minnow, *Gobiocypris rarus* (Teleostei: Cypriniformes). Mitochondrial DNA.

[30] Hua J.H., Han J., Guo Y.Y., Zhou B.S. (2018). Endocrine disruption in Chinese rare minnow (*Gobiocypris rarus*) after long-term exposure to low environmental concentrations of progestin megestrol Check for acetate. Ecotoxicol. Environ. Saf..

[31] Lin Y.S., Wang B., Wang N.H., Ouyang G., Cao H. (2019). Transcriptome analysis of rare minnow (*Gobiocypris rarus*) infected by the grass carp reovirus. Fish Shellfish Immunol..

[32] Hong X.S., Zha J.M. (2019). Fish behavior: A promising model for aquatic toxicology research. Sci. Total Environ..

[33] Zhang J.L., Zhang C.N., Sun P., Shao X. (2016). Tributyltin affects shoaling and anxiety behavior in female rare minnow (*Gobiocypris rarus*). Aquat. Toxicol..

[34] Qiu N., Xu C.S., Wang X.Z., Hou M.M., Xia Z.J., Wang J.W. (2020). Chemicals Weaken Shoal Preference in the Rare Minnow *Gobiocypris rarus*. Environ. Toxicol. Chem..

[35] Qiu N. (2020). Effect of Chemicals Exposure on Behaviors of Rare Minnow. Ph.D. Thesis.

[36] Song C., Liu B.P., Zhang Y.P., Peng Z.L., Wang J.J., Collier A.D., Echevarria D.J., Savelieva K.V., Lawrence R.F. (2017). Modeling consequences of prolonged strong unpredictable stress in zebrafish: Complex effects on behavior and physiology. Prog. Neuro-Psychopharmacol. Biol. Psychiatry.

[37] Collymore C., Tolwani R.J., Rasmussen S. (2015). The Behavioral Effects of Single Housing and Environmental Enrichment on Adult Zebrafish (*Danio rerio*). J. Am. Assoc. Lab. Anim. Sci..

[38] Egan R.J., Bergner C.L., Hart P.C., Cachat J.M., Canavello P.R., Elegante M.F., Elkhayat S.I., Bartels B.K., Tien A.K., Tien D.H. (2009). Understanding behavioral and physiological phenotypes of stress and anxiety in zebrafish. Behav. Brain Res..

[39] Tamilselvan P., Sloman K.A. (2017). Developmental social experience of parents affects behaviour of offspring in zebrafish. Anim. Behav..

[40] Huntingford F.A., Adams C., Braithwaite V.A., Kadri S., Pottinger T.G., Sandoe P., Turnbull J.F. (2006). Current issues in fish welfare. J. Fish Biol..

[41] Wu B.L., Luo S., Xie S.Q., Wang J.W. (2016). Growth of rare minnows (*Gobiocypris rarus*) fed different amounts of dietary protein and lipids. Lab Anim..

[42] Wu B.L., Xiong X.Q., Xie S.Q., Wang J.W. (2016). Dietary lipid and gross energy affect protein utilization in the rare minnow *Gobiocypris rarus*. Chin. J. Oceanol. Limnol..

[43] Wu B.L., Luo S., Wang J.W. (2015). Effects of temperature and feeding frequency on ingestion and growth for rare minnow. Physiol. Behav..

[44] Su L.X., Luo S., Qiu N., Xu C.S., Hou M.M., Xiong X.Q., Wang J.W. (2020). Comparative allometric growth of rare minnow (*Gobiocypris rarus*) in two culture environments. Hydrobiologia.

[45] Luo S., Wu B.L., Xiong X.Q., Wang J.W. (2016). Effects of Total Hardness and Calcium: Magnesium Ratio of Water during Early Stages of Rare Minnows (*Gobiocypris rarus*). Comp. Med..

[46] Selye H. (1950). Stress and the general adaptation syndrome. Br. Med. J..

[47] Barton B.A., Iwama G.K. (1991). Physiological changes in fish from stress in aquaculture with emphasis on the response and effects of corticosteroid. Annual. Rev. Fish Dis..

[48] Oliveira R., Galhardo L. (2009). Psychological stress and welfare in fish. Annu. Rev. Biomed. Sci..

[49] Mommsen T.P., Vijayan M.M., Moon T.W. (1999). Cortisol in teleosts: Dynamics, mechanisms of action, and metabolic regulation. Rev. Fish Biol. Fisher..

[50] Sørensen C., Johansen I.B., Øverli Ø. (2013). Neural plasticity and stress coping in teleost fishes. Gen. Comp. Endocrinol..

[51] Batzina A., Dalla C., Papadopoulou-Daifoti Z., Karakatsouli N. (2014). Effects of environmental enrichment on growth, aggressive behaviour and brain monoamines of gilthead seabream Sparus aurata reared under different social conditions. Comp. Biochem. Physiol. Part A Mol. Integr. Physiol..

[52] Batzina A., Dalla C., Tsopelakos A., Papadopoulou-Daifoti Z., Karakatsouli N. (2014). Environmental enrichment induces changes in brain monoamine levels in gilthead seabream Sparus aurata. Physiol. Behav..

[53] Batzina A., Kalogiannis D., Dalla C., Papadopoulou-Daifoti Z., Chadio S., Karakatsouli N. (2014). Blue substrate modifies the time course of stress response in gilthead seabream Sparus aurata. Aquaculture.

[54] Rosengren M., Kvingedal E., Naslund J., Johnsson J.I., Sundell K. (2016). Born to be wild: Effects of rearing density and environmental enrichment on stress, welfare, and smolt migration in hatchery-reared Atlantic salmon. Can. J. Fish. Aquat. Sci..

[55] Cogliati K.M., Herron C.L., Noakes D.L.G., Schreck C.B. (2019). Reduced stress response in juvenile Chinook Salmon reared with structure. Aquaculture.

[56] Naslund J., Rosengren M., Del Villar D., Gansel L., Norrgard J.R., Persson L., Winkowski J.J., Kvingedal E. (2013). Hatchery tank enrichment affects cortisol levels and shelter-seeking in Atlantic salmon (*Salmo salar*). Can. J. Fish. Aquat. Sci..

[57] Keck V.A., Edgerton D.S., Hajizadeh S., Swift L.L., Dupont W.D., Lawrence C., Boyd K.L. (2015). Effects of habitat complexity on pair-housed zebrafish. J. Am. Assoc. Lab. Anim. Sci..

[58] Dickens M.J., Romero L.M.A. (2013). Consensus endocrine profile for chronically stressed wild animals does not exist. Gen. Comp. Endocrinol..

[59] Koob G.F. (1996). Hedonic valence, dopamine and motivation. Mol. Psychiatry.

[60] Phillips A.G., Vacca G., Ahn S.A. (2008). Top-down perspective on dopamine, motivation and memory. Pharmacol. Biochem. Behav..

[61] Beninger R.J. (1983). The role of dopamine in locomotor activity and learning. Brain Res. Rev..

[62] Salamone J.D. (1992). Complex motor and sensorimotor functions of striatal and accumbens dopamine: Involvement in instrumental behavior processes. Psychopharmacology.

[63] Saudou F., Amara D.A., Dierich A., Lemeur M., Ramboz S., Segu L., Buhot M.C., Hen R. (1994). Enhanced aggressive-behavior in mice lacking 5-HT1B receptor. Science.

[64] Mann J.J. (1999). Role of the serotonergic system in the pathogenesis of major depression and suicidal behavior. Neuropsychopharmacology.

[65] Pang T.Y.C., Du X., Zajac M.S., Howard M.L., Hannan A.J. (2009). Altered serotonin receptor expression is associated with depression-related behavior in the R6/1 transgenic mouse model of Huntington’s disease. Hum. Mol. Genet..

[66] Paterson N.E., Markou A. (2007). Animal models and treatments for addiction and depression co-morbidity. Neurotoxic. Res..

[67] Mann J.J., Bloom F.E., Kupfer D.J. (1995). Violence and aggression. Psychopharmacology: The Fourth Generation of Progress.

[68] Benaroya-Milshtein N., Hollander N., Apter A., Kukulansky T., Raz N., Wilf A., Yaniv I., Pick C.G. (2004). Environmental enrichment in mice decreases anxiety, attenuates stress responses and enhances natural killer cell activity. Eur. J. Neurosci..

[69] Gortz N., Lewejohann L., Tomm M., Ambree O., Keyvani K., Paulus W., Sachser N. (2008). Effects of environmental enrichment on exploration, anxiety, and memory in female TgCRND8 Alzheimer mice. Behav. Brain Res..

